# Years of life lost methods must remain fully equitable and accountable

**DOI:** 10.1007/s10654-022-00846-9

**Published:** 2022-03-04

**Authors:** Grant M. A. Wyper, Brecht Devleesschauwer, Colin D. Mathers, Scott A. McDonald, Niko Speybroeck

**Affiliations:** 1 Place and Wellbeing Directorate, Public Health Scotland, Glasgow, Scotland, UK; 2 Department of Translational Physiology, Infectiology and Public Health, Ghent University, Merelbeke, Belgium; 3 Department of Epidemiology and Public Health, Sciensano, Brussels, Belgium; 4 Consultant on Global Health, Geneva, Switzerland; 5 College of Medicine and Veterinary Medicine, University of Edinburgh, Edinburgh, Scotland; 6 Centre for Infectious Disease Control, National Institute for Public Health and the Environment (RIVM), Bilthoven, The Netherlands; 7 Research Institute of Health and Society (IRSS), Université Catholique de Louvain, Brussels, Belgium

How to estimate population health loss due to COVID-19 has been greatly contested [1]. This has resulted in various approaches being applied to estimate the years of life lost to premature mortality (YLL), which Ferenci discusses in detail [2]. Due to the overwhelming impact of the COVID-19 pandemic, YLL methods have become more frequently used and sometimes, unfortunately, misused.

Although the YLL measure has been around since the 1940s, the creation of the Global Burden of Disease study and its associated methodological developments have increased awareness and understanding over its application, although many issues are still challenged [3]. Firstly, it is important to articulate that 'true' YLL can never be observed, and as such, language that indicates that YLL estimates have been under- or over-estimated may be misplaced. From the perspective of informing public health policy, the counterfactual to be applied in the estimation of YLL is that of an ideal, aspirational, standard based upon desirably low mortality risks. The merits of resulting YLL estimates should be appraised entirely on their data inputs and choice of age-conditional life expectancy valuation.

Secondly, the key utility of YLL estimates lays in comparisons, whether with respect to other health outcomes, across time, or between demographic sub-populations or geographic regions. This requires that the same measure of loss of life years be used for a death at a given age, whatever the cause, the subpopulation in which the death occurs, or the time period in which the death occurs. Therefore, one cannot arbitrarily decide that selected causes will be corrected for co-morbidities, as that will impact the validity of any resulting comparison across causes, populations or time periods. It also imposes an enormous and generally unrealistic demand for data on the distribution of all relevant comorbidities in the people who die of COVID-19, presumably stratified by country and time period as well as on the counterfactual (unobservable) risks of death in the absence of COVID-19. And in the absence of data, additional assumptions would be required. Furthermore any proposals for adjustment would also need to be considered from the alternative perspective, that being that a non-COVID-19 death could be causally related to a prior COVID-19 infection. From this perspective, data availability would be even more likely to be sparser.

COVID-19 has represented a novel mortality hazard, and researchers have been trying to apply methods to assess its impact on population health. However, although COVID-19 is novel, the methodological situation to estimating YLL in situations of sudden heightened mortality risk is not. The same is true for most sudden spikes in mortality risk that occurs. For example, in individuals with severe pneumonia, or for those suffering a severe road traffic accident, the risk of death would be expected to be greater in those whose health is impaired, compared to those in excellent health. Should a fatal road traffic accident result in fewer YLL in a frail citizen, compared to someone of the same age without underlying health issues? The answer to this is no, because we are describing health outcomes, which on their own cannot capture the accumulation of risks and occurrence of other health outcomes along the life course.

It is easy to understand why a ‘correction’ for co-morbidities could offer a plausible solution. However, this ignores the already well-established causal linkage of risk factor exposures to health outcomes. From this perspective—rather than adjusting YLL—one can see that health outcomes can potentially be attributed to one or more risk factors. This would be the case for COVID-19, given that those with specific co-morbidities are at higher risk of death. There is overwhelming evidence which makes clear the causal relationships of these co-morbidities with prior risk factor exposure, and how inequalities in these exposure result in inequalities in causally-related health outcomes. Ferenci has concluded that YLL due to COVID-19 is likely to be 12% lower when accounting for multimorbidity. To give a sense of scale of the impact of attributing YLL to risk factor exposure, the GBD 2019 study reports that the risk factor attributable YLL for lower respiratory infections in the World Health Organization’s European region is 62% [4]. This highlights the highly selective nature of adjusting for prior co-morbidities, and would massively overestimate the COVID-19 unattributable YLL, which is counterintuitive to the prior hypothesis which Ferenci raises. The attributable YLL associated with a risk factor exposure are generally calculated by comparison with a counterfactual risk exposure scenario (usually zero risk or minimum risk) by adding the YLL across specific causes of death in each scenario and subtracting the counterfactual total from the total in the exposed population. If part of the excess mortality risk associated with some exposures associated with comorbid diseases is already adjusted out of the cause-specific YLL, it becomes impossible to estimate the actual total attributable YLL for risk factors in any comparable way.

Attempts to treat cause-specific YLL estimates differently—by selective adjustment—would mean we lose a focus of the environments and risks that are responsible for that death occurring in the first place [5]. Summary indicators of population health, such as YLL, are important for informing debates on public health policy action. Adjustments for comorbidity would likely lead to uncomfortable ethical outcomes. Many socioeconomically disadvantaged regions, or indeed within-country sub-populations, suffer higher rates of morbidity than their counterparts meaning that they would likely be subject to larger downwards adjustment, even though they have greater health needs. This would lead to uncomfortable ethical outcomes, as socioeconomically disadvantaged regions could be disregarded for resource-limited interventions over their more advantaged counterparts, many of which are already privileged, because the members of the former have ‘less to gain’. These approaches should be avoided when aiming to detect unfair, and unjust, inequalities in population health.
